# Supercritical CO_2_ Extraction of Terpenoids from *Indocalamus latifolius* Leaves: Optimization, Purification, and Antioxidant Activity

**DOI:** 10.3390/foods13111719

**Published:** 2024-05-30

**Authors:** Yadan Chen, Yanbin Wang, Liang He, Liling Wang, Jianchen Zhao, Zhenya Yang, Qin Li, Rui Shi

**Affiliations:** 1 The Key Laboratory of State Forest Food Resources Utilization and Quality Control, Department of Forest Foods, Zhejiang Academy of Forestry, Hangzhou 310023, China; 2 Department of Food Science and Technology, College of Light Industry and Food Engineering, Nanjing Forestry University, Nanjing 210037, China

**Keywords:** *Indocalamus latifolius*, supercritical CO_2_ (SC-CO_2_), optimization, GC-MS, purification, antioxidant, oxidative stress

## Abstract

This study aimed to investigate the efficacy of supercritical CO_2_ (SC-CO_2_) extraction in enhancing the extraction rate, purity, and antioxidant activity of *Indocalamus latifolius* (Keng) McClure (Poaceae) leaf terpenoids (ILLTs). Crude extracts obtained from leaves were subjected to qualitative and quantitative analyses, revealing neophytadiene, phytol, β-sitosterol, β-amyrone, squalene, and friedelin as the primary terpenoid constituents, identified through gas chromatography–mass spectrometry (GC-MS). Compared with steam distillation extraction (SD), simultaneous distillation extraction (SDE), ultra-high pressure-assisted n-hexane extraction (UHPE-Hex), ultra-high pressure-assisted ethanol extraction (UHPE-EtOH), ultrasound-assisted n-hexane extraction (UE-Hex), and ultrasound-assisted ethanol extraction (UE-EtOH), SC-CO_2_ exhibited a superior ILLT extraction rate, purity, and antioxidant activity. Scanning electron microscopy (SEM) observations of the residues further revealed more severe damage to both the residues and their cell walls after SC-CO_2_ extraction. Under optimal parameters (4.5 h, 26 MPa, 39 °C, and 20% ethyl alcohol), the ILLT extraction rate with SC-CO_2_ reached 1.44 ± 0.12 mg/g, which was significantly higher than the rates obtained by the other six methods. The subsequent separation and purification using WelFlash C18-l, BUCHI-C18, and Sephadex LH-20 led to an increase in the purity of the six terpenoid components from 12.91% to 93.34%. Furthermore, the ILLTs demonstrated cytotoxicity against HepG2 cells with an IC_50_ value of 148.93 ± 9.93 μg/mL. Additionally, with increasing concentrations, the ILLTs exhibited an enhanced cellular antioxidant status, as evidenced by reductions in both reactive oxygen species (ROS) and malondialdehyde (MDA) levels.

## 1. Introduction

*Indocalamus latifolius* (Keng) McClure (Poaceae) is native to China, and is renowned for its distinctive pleasant aroma [1]. Due to their broad leaves, they are frequently utilized as packaging materials during the traditional Chinese Dragon Boat Festival. Studies have demonstrated that these leaves possess medicinal properties such as heat-clearing, hemostatic, detoxifying, and anti-inflammatory effects [2]. Previous phytochemical studies on *I*. *latifolius* leaves have isolated and identified active polysaccharides, flavonoids [3], terpenoids, and other secondary metabolites [1]. Terpenoids, a super family of structurally diverse natural products [4], are found in animals, plants, fungi, algae, corals, and others, which play an important role in antiviral activity [5], anti-inflammatory effects [6], antitumor activity [7], and antifungal activity [8], and have garnered significant attention. However, there has been no systematic investigation into the various types of diterpenes that can be isolated from *I*. *latifolius* leaves, and the physicochemical properties of the terpenoids derived from these leaves remain poorly understood. 

Given the impact of the extraction process, the pre-treatments, and the choice of solvent on the chemical composition and biological activities of the extracts [9], selecting an optimization system to enhance yield is crucial. Prolonged extraction times associated with conventional methods (such as stirring, heat reflux, or a Soxhlet apparatus) result in increased degradation of thermolabile and photosensitive compounds [10]. Supercritical carbon dioxide (SC-CO_2_) is the most common solvent utilized in supercritical fluid extraction, offering numerous advantageous characteristics including enhanced selectivity, a greater purity of extracts [11], preservation of heat-sensitive compounds and natural flavors, and it is an environmentally friendly technology [12]. This method has been widely employed for the extraction and enrichment of flavonoids [13], phenolic compounds [14], phytosterols [15], polysaccharides [16], and diterpenes [10]. Barbosa et al. [17] utilized SC-CO_2_ to extract essential oils, including kahweol, cafestol, and 16*-O-*methylcafestol from spent coffee grounds, resulting in a 212–410% increase in diterpene concentration. According to the research of Glisic et al. [18], a lower pressure and reduced CO_2_ consumption were found to be more conducive to the extraction of low molecular weight compounds. 

Currently, SC-CO_2_ has been applied to extract polyphenols from bamboo leaves [19]. However, there is a scarcity of studies investigating the extraction process, cytotoxicity, and cellular antioxidant capacity of *I*. *latifolius* leaf terpenoids (ILLTs). Thus, this study aimed to identify the most suitable extraction method for ILLTs, comparing the extraction rates, purities, and extract antioxidant activities of different extraction methods. The optimization of extraction conditions was conducted using single-factor and response surface experimental methods to establish a green extraction process for ILLTs, aiming to preserve the activity of terpenoid compounds and achieve high extraction rates. Under the optimal conditions, crude extracts of *I*. *latifolius* leaves were obtained and subsequently purified to obtain terpenoid extracts with a higher purity. Finally, the antiproliferative activity of the ILLTs on HepG2 cells and their protective ability against TBHP-induced oxidative damage in HepG2 cells were investigated.

## 2. Materials and Methods

### 2.1. Materials and Chemicals

*I*. *latifolius* leaves, identified by researcher Liu Bentong, were gathered from the Bamboo Botanical Garden of Zhejiang Academy of Forestry. Carbon dioxide (CO_2_, 99.0% purity) was purchased from Hangzhou Yuetong Co., Ltd. (Hangzhou, China); petroleum ether, dichloromethane, isopropyl alcohol, methanol, ethyl acetate (HPLC grade), and DPPH were bought from Xilong Scientific Co., Ltd. (Shantou, China) WelFlash C18-l, C18 Bio-sep, and Sephadex LH-20 were bought from Welch Materials (Shanghai) Co., Ltd. (Shanghai, China)Standards for phytol and β-sitosterol (≥95% purity) were delivered from Shanghai Acmec Biochemical Co., Ltd. (Shanghai, China). The standard for β-amyrone (98% purity) was purchased from J&K Scientific Co., Ltd. (San Jose, CA, USA) Standards for friedelin, squalene, neophytadiene (≥90% purity), and Vc were purchased from Aladdin Biochemical Technology Co., Ltd. (Shanghai, China) Dulbecco’s modified Eagle’s medium (DMEM), penicillin–streptomycin mixed solution (10,000 units/mL and 10 mg/mL, respectively), trypsin–EDTA, fetal bovine serum (FBS), and the human hepatoma carcinoma HepG2 cell line were bought from Shanghai Fuheng Biotechnology Co., Ltd. (Shanghai, China). Cell counting kit-8 (CCK-8) and test kits were purchased from Beyotime Biotechnology (Shanghai, China). TBHP was obtained from Alfa Aesar (Shanghai, China).

### 2.2. GC-MS Analysis

A gas chromatography–mass spectrometry (GC-MS) apparatus (GCMS-QP2020 NX, Shimadzu Global Laboratory Consumables Co., Tokyo, Japan) was used to analyze the crude extract and diterpene content (neophytadiene, phytol, β-sitosterol, β-amyrone, squalene, and friedelin). The chromatographic column employed was an SH-I-5il MS (30 m × 0.25 mm × 0.25 μm film thickness), with high-purity helium used as the gas carrier at a flow rate of 1 mL/min. The injector temperature was maintained at 250 °C. The injection volume was 1.0 mL with unsplit stream sampling. The temperature program commenced at 50 °C (held for 1 min), subsequently ramping up to 160 °C at a rate of 8 °C per minute (held for 1 min), and then ramping up to 300 °C at a rate of 5 °C per minute (held for 30 min). The MS ion source temperature was maintained at 230 °C, while the inlet line temperature was set to 280 °C. The scan range encompassed 35–600 amu at an electron energy of 70 eV, with a solvent delay of 4 min. The obtained chromatographic ion fragmentation maps were cross-referenced with ion fragmentation in NIST 20 (National Institute of Standards and Technology) for target compound characterization.

### 2.3. Quantification of Diterpenes

Quantification of the diterpenes: Neophytadiene, phytol, β-sitosterol, β-amyrone, squalene, and friedelin were dissolved and diluted with methyl alcohol to form 2.5 μg/mL, 5 μg/mL, 10 μg/mL, 20 μg/mL, and 40 μg/mL standard solutions, which were then analyzed by GC-MS as detailed in Section 2.2 after passing through a 0.45 mm nylon membrane. The standard curve for diterpenes was calculated as follows:

Phytol: y = 1 × 10^8^x − 69,112 (R^2^ = 0.9995); neophytadiene: y = 5 × 10^8^x − 67,1704 (R^2^ = 0.9992); β-sitosterol: y = 15,423x − 2466.6 (R^2^ = 0.9997); β-amyrone: y = 1 × 10^8^x − 90,468 (R^2^ = 0.9992); friedelin: y = 3 × 10^7^x − 8826.5 (R^2^ = 0.9996); squalene: y = 7 × 10^8^x − 28,5411 (R^2^ = 0.9996).

In addition, the absolute content of other compounds was based on the content of phytol (m_p_), the relative content of phytol (C_P_), and the relative content of compounds (C_X_) detected by GC-MS. The formula is as follows:

(1) $$\mathrm{Absolute}\;\mathrm{content}\;\mathrm{of}\;X\;\left(\mathrm{mg}\right)=\frac{m_{p}{\;\times \;C}_{X}}{C_{p}}$$

### 2.4. Extraction Methods

#### 2.4.1. Supercritical Carbon Dioxide Extraction (SC-CO_2_)

The experimental assays were performed using supercritical extracting equipment (Spe-ed SFE, Applied Separations Inc., Allentown, PA, USA). The extractor was packed with 20 g of *I*. *latifolius* leaf powder. The thermostatic bath was adjusted to 5 ± 1 °C and the pressure was adjusted by pumping supercritical carbon dioxide (SC-CO_2_). The product separated from the fluid was collected into amber-colored glass bottles. After dissolving in 10 mL of an ethanol solution, the product was passed through a 0.45 μm organic filter membrane and analyzed by GC-MS.

#### 2.4.2. Steam Distillation (SD)

Approximately 40 g powder of *I*. *latifolius* leaf powder was added to 500 mL of distilled water, followed by 4 h of distillation. The distillation process was repeated twice using n-hexane. The n-hexane solution was collected and rotary evaporated until the solvent completely evaporated. The residue was weighed, dissolved in 5 mL of n-hexane, and treated with anhydrous sodium sulfate. Then, it was filtered through a 0.45 μm organic filter membrane and analyzed by GC-MS.

#### 2.4.3. Simultaneous Distillation Extraction (SDE)

Approximately 40 g powder of *I*. *latifolius* leaf powder and 500 mL of distilled water were added to a 1000 mL round-bottom flask, which was placed in the light phase end of a simultaneous distillation extraction device with electric heat jacket heating. On the other side, 50 mL of dichloromethane was placed in the thermostatic bath (45 ± 1) °C. After 2.5 h, the mixture was rotary evaporated at 30 °C until the dichloromethane completely evaporated. The residue was weighed, dissolved in 5 mL of n-hexane, and treated with anhydrous sodium sulfate. Then, it was passed through a 0.45 μm organic filter membrane and analyzed by GC-MS. 

#### 2.4.4. Ultrasound-Assisted Extraction (UE)

Approximately 20 g of *I*. *latifolius* leaf powder was placed in a conical flask and 200 mL of n-hexane or ethanol was added. The temperature was set to 40 °C, and ultrasound was applied (25/45 kHz, 600 W) for 20 min using an ultrasonic cleaning machine (SB25-12DTS, Nanjing Safer Biotech Co., Nanjing, China). The supernatant was collected, and this process was repeated three times. After the third ultrasound extraction, the mixture was centrifuged at 3000 rpm for 10 min at room temperature, and the supernatant was collected. The supernatants were combined, rotary evaporated at 30 °C until the solvent completely evaporated, and the residue was weighed. Then, 10 mL of n-hexane was added, and an appropriate amount of anhydrous sodium sulfate was added. The solution was filtered through a 0.45 μm organic membrane filter and analyzed by GC-MS.

#### 2.4.5. Ultra-High Pressure-Assisted Extraction (UHPE)

Approximately 20 g of *I*. *latifolius* leaf powder and 500 mL of an n-hexane or ethanol solution were placed in a vacuum-sealed bag. The sealed bag was placed in the processing chamber of an ultra-high-pressure apparatus and treated at 400 MPa for 10 min. After centrifugation at 3000 rpm for 10 min at room temperature, the supernatant was collected and rotary evaporated until the solvent evaporated completely. The residue was weighed, and then 10 mL of n-hexane was added along with anhydrous sodium sulfate. The solution was filtered through a 0.45 μm organic membrane filter and analyzed by GC-MS.

### 2.5. Measurement of Scanning Electron Microscopy (SEM)

The morphological changes of *I*. *latifolius* leaves before and after the treatments were observed by scanning electron microscopy (S–3400 N, HITACHI, Tokyo, Japan) at 12.5 kV. Images of typical morphologies were captured at 500× magnification.

### 2.6. Determination of In Vitro Antioxidant Activity of ILLTs by Different Methods

#### 2.6.1. DPPH Radical Scavenging Activity Assay

Following the method of reference [20] with modifications, ILLT crude extracts from the different extraction methods were prepared into sample solutions with concentrations ranging from 0.4 to 1.2 mg/mL. After mixing according to Table 1, the mixture was placed in the dark for 30 min, and the absorbance was measured at 517 nm using a spectrophotometer. Vc was used as a positive control. The DPPH radical scavenging activity was calculated according to Formula (2):

(2) $$\mathrm{DPPH}\;\mathrm{radical}\;\mathrm{scavenging}\;\mathrm{activity}\;\left(\%\right)=\left[1\;-\frac{\left(A_{\mathrm{sample}}-{\;A}_{\mathrm{blank}}\right)}{A_{\mathrm{control}}}\right]\;\times \;100$$

#### 2.6.2. Hydroxyl Radical Scavenging Activity Assay

ILLT crude extracts from the different extraction methods were prepared into sample solutions with concentrations ranging from 0.4 to 1.2 mg/mL. Then, the samples (1 mL) were incubated with a salicylic acid ethanol solution (1 mL, 9.0 mmol/L), FeCl_2_ (1 mL, 9.0 mmol/L), and H_2_O_2_ (1 mL, 8.8 mmol/L) for 30 min at 37 °C in the dark. The absorbance of the reaction mixture was measured at 536 nm using the multifunctional enzyme marker (Varioskan Flash, Thermo Fisher Scientific, Waltham, MA, USA). The hydroxyl radical scavenging rate was calculated based on Equation (3):

(3) $$\mathrm{Hydroxyl}\;\mathrm{radical}\;\mathrm{scavenging}\;\mathrm{activity}\;\left(\%\right)=\left[1\;-\frac{\left(A_{1}-{\;A}_{2}\right)}{A_{0}}\right]\;\times \;100$$

where A_0_ is the absorbance of the control sample, A_1_ is the absorbance of the blank sample prepared by replacing nanoliposome samples with ultrapure water, and A_2_ is the absorbance of the sample prepared without adding salicylic acid ethanol solution.

### 2.7. Optimization of SC-CO_2_ Extraction

#### 2.7.1. Single-Factor Test

The effects of four different factors on diterpene extraction were investigated, which were the extraction stress (10, 20, 30, 40, and 50 MPa), extraction temperature (32, 37, 42, 47, and 52 °C), cosolvent concentration (0, 10, 20, 30, and 40% *v*/*w*), and extraction time (1, 2, 3, 4, 5, and 6 h). The average value was tested three times to determine the appropriate experimental conditions.

#### 2.7.2. Optimization of Experimental Design

RSM based on the Box–Behnken design was applied to evaluate the effect of the four variables (extraction stress, extraction temperature, extraction time, and cosolvent) on the response of ILLT yield. According to the results of the single-factor experiment, a central composite design with three levels of each factor was utilized to optimize the SC-CO_2_ extraction conditions (Table 2).

### 2.8. Calculation of Diterpene Yield and Purity

The diterpene yield and purity were quantified according to the Formulas (4) and (5) based on the mass ratio between the extract (M_ext_) and raw material (M) and between diterpenes (M_ITLD_) and the products (M_pur_).

(4) $$\mathrm{Diterpene}\;\mathrm{Yield}\;\left(\mathrm{mg}/g\right)=\frac{M_{\mathrm{ext}}}{M}\;\times \;100$$

(5) $$\mathrm{Diterpene}\;\mathrm{Purity}\;\left(\%\right)=\frac{M_{\mathrm{ITLD}}}{M_{\mathrm{pur}}}\;\times \;100$$

### 2.9. Isolation and Purification

Using crude extracts from SC-CO_2_ as raw materials, the method referred to in references [21,22,23] was employed for the isolation and purification with adjustments. The steps were as follows: (1) The crude extract was dissolved in n-hexane at a mass/volume ratio of 1:10. After filtration through a 0.45 μm organic membrane, the filtrate was collected and rotary evaporated until the solvent completely evaporated. Then, petroleum ether (60–90 °C) was added to ensure complete dissolution. (2) The above petroleum ether extract was separated by WelFlash C18-l column chromatography using petroleum ether/dichloromethane (7:3) as the eluent, obtaining 20 fractions. Each fraction was concentrated and analyzed by GC-MS. Fractions containing a higher content of triterpenoids were combined and concentrated. (3) The concentrated solution obtained above was chromatographed on a BUCHI-C18 column using a gradient elution of petroleum ether/isopropanol (94:6-88:12-75:25), yielding 12 fractions. After concentration, each fraction was weighed and analyzed by GC-MS. The purity was calculated using the differential method, and fractions with a higher purity were collected. (4) The above fractions were concentrated and further purified using Sephadex LH-20 chromatography (70% MeOH/ethyl acetate, 4:1-1:1-1:4), resulting in 25 fractions (F1-25). The purity of each fraction was calculated using the same method as above, and each fraction was analyzed by GC-MS.

### 2.10. Cell Culture

HepG2 cells were cultured in DMEM, supplemented with 10% FBS and 1% penicillin and streptomycin. The cells were maintained within a humidified cell incubator at 37 °C and 5% CO_2_ atmosphere.

#### 2.10.1. Cell Viability

Cell viability was assessed using the CCK-8 assay [24]. The cells were seeded at a density of 1 × 105 cells per well within 96-well plates for 24 h. Subsequently, the cells were exposed to the ILLTs (80–250 μg/mL) and a TBHP solution (5–100 μg/mL) for 24 h. The culture medium was removed from each well, and the treated cells underwent two washes with PBS. A serum-free DMEM medium (90 mL) and CCK-8 solution (10 mL) were introduced to each well. The cultures were then incubated for an additional 0.5 h at 37 °C. Using a multifunctional enzyme marker (Varioskan Flash, Thermo Fisher Scientific), the absorbance was measured at 450 nm. Cell viability was calculated using Formula (6):

(6) $$\mathrm{Cell}\;\mathrm{viability}\;(\%)=\left(\frac{A_{1}-{\;A}_{0}}{A_{2}-{\;A}_{0}}\right)\;\times \;100$$

where A_0_ is the absorbance of the blank group, A_1_ is the absorbance of the treated group, and A_2_ is the absorbance of the control group.

#### 2.10.2. Determination of ROS and MDA Levels in Cells

The contents of ROS and MDA were determined as described by Wang et al. [25] with slight modifications. Firstly, digested HepG2 cells were inoculated at a density of 1 × 105 cells/well in 96-well cell culture plates featuring a black periphery and transparent bottom for 24 h. The initial culture medium was discarded. Then, 80–90 μg/mL of the ILLTs was introduced into the wells. After 24 h, this was replaced with 5 g/mL of a TBHP solution for another 24 h. Subsequently, 10 µM DCFH-DA was added and the plate was incubated for 0.5 h, followed by a PBS wash. Utilizing a multifunctional microplate reader, the fluorescence intensity of the 96-well plate was measured with excitation at 485 nm and emission at 528 nm. Intracellular ROS levels in the experimental group were expressed as a percentage of the negative control group and were calculated according to Formula (7):

(7) $$\mathrm{ROS}\;\mathrm{level}\;\left(\%\right)=\frac{F}{F_{0}}\;\times \;100$$

where F is the fluorescence intensity of the injury model group or drug intervention group, and F_0_ is the fluorescence intensity of the blank control group.

For the assessment of the MDA content, digested HepG2 cells (6 × 10^5^) were separately pipetted into 6-well cell culture dishes and cultured for 24 h. After following the above steps, the MDA content was measured using the corresponding test kits.

#### 2.10.3. The Production of ROS Was Observed by Microscopy

The digested cells were inoculated into 6-well plates at a density of 3 × 10^5^ cells per well and incubated for 24 h. The subsequent steps were described in Section 2.10.2. The observation of fluorescence production within the cells was performed using a fluorescence microscope (DM6B, Leica, Heidelberg, Germany) equipped with a FITC fluorescence channel.

### 2.11. Statistical Analysis

All data were reported as the mean ± standard deviation, with each experiment repeated three times. Statistical analyses were performed using SPSS software (Versions 19, Armonk, NY, USA) and Origin 2022 (Origin Lab Co., Northampton, MA, USA). To assess differences, one-way analysis of variance (ANOVA) and Duncan’s multiple comparisons were conducted. The different letters indicate significant differences (*p* < 0.05) among samples within the same experimental group.

## 3. Results and Discussion

### 3.1. Impact of Different Extraction Methods on the Composition of I. latifolius Leaf Extracts

**Figure 1:**
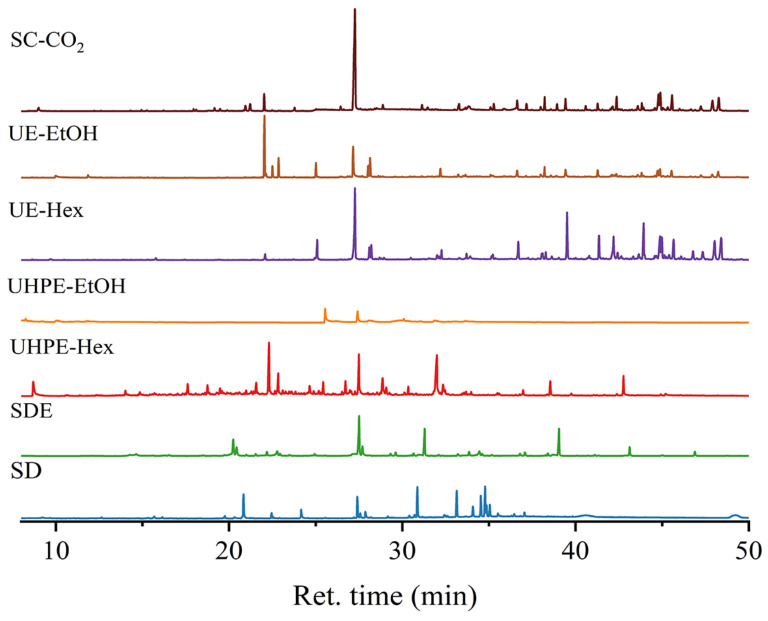
Total ion compound chromatograms (TICs) of *I. latifolius* leaf extracts.

Various extraction methods, such as SD, SDE, UHPE-Hex, UHPE-EtOH, UE-Hex, UE-EtOH, and SC-CO_2_, were utilized for the extraction of terpenoids from *I*. *latifolius* leaves. The total ion chromatogram is depicted in Figure 1, while Table 3 illustrates the composition of the extracts, identifying a total of 111 components. The total absolute contents of the components were identified as follows: 10.360 mg (SD), 7.347 mg (SDE), 30.686 mg (UHPE-Hex), 4.682 mg (UHPE-EtOH), 13.749 mg (UE-EtOH), 35.957 mg (UE-Hex), and 28.160 mg (SC-CO_2_). 

The GC-MS analysis of the UHPE-EtOH extract revealed the presence of only seven compounds, suggesting that the ethanol–ultrasound conditions might not be optimal for extracting active ingredients from *I*. *latifolius* leaves. Conversely, SD, SDE, and UHPE-Hex yielded relatively fewer components, with 18, 28, and 24 compounds, respectively, predominantly comprising terpenoids and hydrocarbons. The UE-Hex, UE-EtOH, and SC-CO_2_ extraction methods yielded 42, 27, and 38 components, respectively, predominantly terpenoids, including phytol, neophytadiene, β-amyrone, squalene, friedelin, β-sitosterol, campesterol, stigmasterol, sclareol, α-ionone, D-friedoolean-14-en-3-one, phytone, β-ionone, lup-20(29)-en-3-one, vitamin E, and γ-tocopherol. However, the UE extracts contained a large number of ester compounds. 

**Figure 2:**
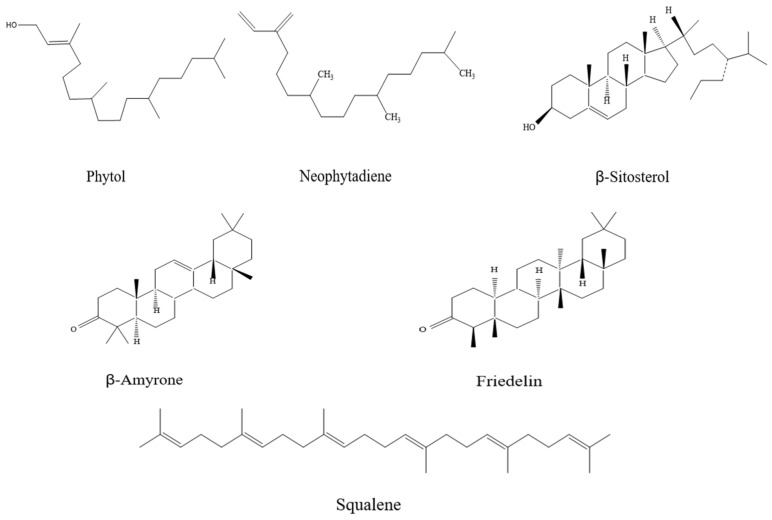
The chemical structures of terpenoids.

The number and total absolute content of terpenoids in the SC-CO_2_ extracts (16, 25.207 mg) were significantly higher than those in the UE-Hex (12, 23.880 mg) and UE-EtOH (13, 9.665 mg) extracts. Notably, neophytadiene, phytol, β-sitosterol, β-amyrone, squalene, and friedelin exhibited relatively high contents in both the SC-CO_2_ and ultrasound extracts. The chemical structures of these compounds are depicted in Figure 2. Thus, these six terpenoid compounds were regarded as the primary terpenoids in *I*. *latifolius* leaves.

### 3.2. Influence of Different Extraction Methods on the Extraction Rate of I. latifolius Leaf Terpenoids (ILLTs)

**Figure 3:**
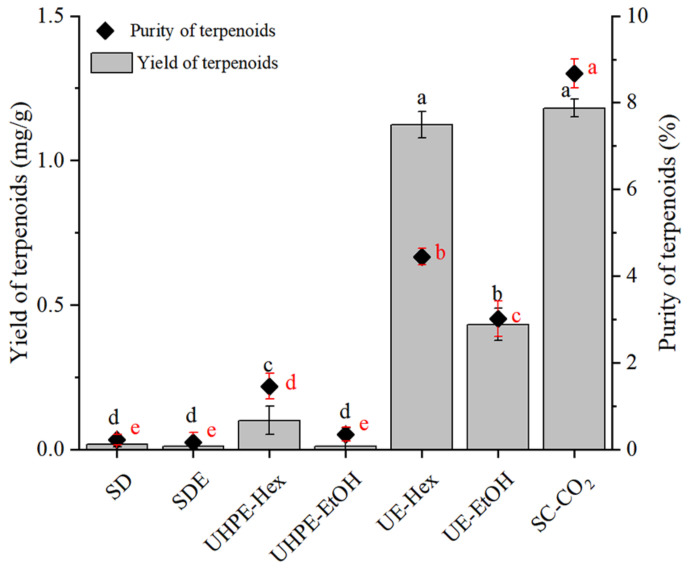
Main effect plot showing the effect of each factor on the yield (mg/g). Different letters indicate significant differences (*p* < 0.05). The black and red letters for the statistical analysis represent the significance of the extraction rate and purity differences, respectively.

The samples underwent GC-MS analysis to determine the contents of neophytadiene, phytol, β-sitosterol, β-amyrone, squalene, and friedelin, which were calculated using standard curves. Figure 3 displays the extraction rate and purity of the ILLTs using seven different extraction methods (SD, SDE, UHPE-Hex, UHPE-EtOH, UE-Hex, UE-EtOH, and SC-CO_2_). As shown in the figure, there were significant differences in the total extraction rate and purity of the six terpenoids obtained by different extraction methods. The SC-CO_2_ extraction rate exhibited the highest extraction yield and purity (1.18 ± 0.03 mg/g ILLTs, 8.67 ± 0.23%), followed by UE-Hex (1.12 ± 0.05 mg/g ILLTs, 4.44 ± 0.19%) and UE-EtOH (0.43 ± 0.06 mg/g ILLTs, 3.02 ± 0.19%). Tang et al. [26] discovered that supercritical fluid extraction technology was beneficial for the extraction of terpenoids. Moreover, the studies of He et al. [27] and Glisic et al. [28] demonstrated that compared to ultrasound-assisted extraction, supercritical extraction can obtain higher extraction rates and purity of flavonoids or essential oils from plants. The extraction efficiency of bioactive components is correlated with solvent polarity [29]. Ethanol, used as a solvent in ultrasound-assisted extraction, exhibited a better extraction efficiency than n-hexane, albeit with inferior purity. Luca et al. [30] and Zhang et al. [31] reported that an ethanol solution aids in extracting total triterpenoids, and it also facilitates the dissolution of other active ingredients. Although the extraction rates of SC-CO_2_ and UE-Hex were similar, SC-CO_2_ exhibited a significantly higher purity. Additionally, both UE and UHPE suffer from drawbacks such as high consumption of organic solvents, solvent residues, and increased impurities, thereby complicating subsequent purification steps. The lower extraction rates of the two distillation methods might result from compound decomposition during prolonged high-temperature processes and large polarity differences [32].

### 3.3. SEM Imaging of I. latifolius Leaves during Extraction Using Different Extraction Methods

**Figure 4:**
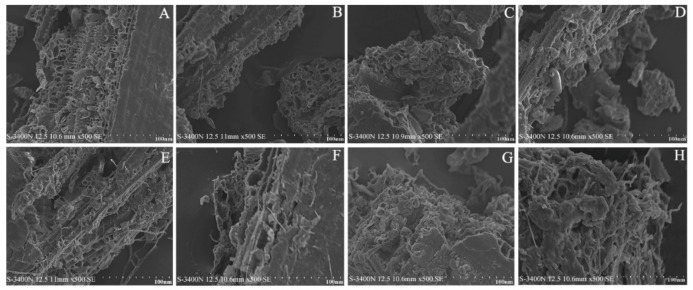
SEM of *I. latifolius* leaf powder before (**A**) and after extraction by SD (**B**), SDE (**C**), UHPE-EtOH (**D**), UHPE-Hex (**E**), UE-EtOH (**F**), UE-Hex (**G**), and SC-CO_2_ (**H**).

SEM can be employed to observe the microstructural changes of *I*. *latifolius* leaves following extraction using the various extraction methods to elucidate the impact of these methods on the physical structure of the leaves. As depicted in Figure 4A, the surface of the leaves prior to the treatment appeared smooth, with regular microcavity structures visible along the edges. In Figure 4B–D, leaf residues treated by SD, SDE, and UHPE-EtOH demonstrated partially regular microcavity structures, exhibiting relatively smooth surfaces and fewer cracks. Figure 4E–H illustrate the residues treated by UHPE-Hex, UE-EtOH, UE-Hex, and SC-CO_2_, respectively, displaying rough surfaces, loose structures, and notable cracks and fragments. The employment of the UE and SC-CO_2_ extraction methods, particularly SC-CO_2_, led to more severe fragmentation of the leaves, resulting in greater damage to the cell walls and enhanced release of terpenoid compounds [33]. Ultrasonic action can enhance the cavitation effect of the liquid medium, thereby increasing the diffusion coefficient of the solutes (active ingredients) [34]. In addition, organic solvents have certain dissolution and softening effects on the cell wall structure, exacerbating the damage to microcavities [35]. The SEM results further supported the effectiveness of the UE and SC-CO_2_ extraction methods in promoting the yield of terpenoid compounds from *Indocalamus latifolius* leaves.

### 3.4. Study on the Antioxidant Activity of Extracts by Different Methods

**Figure 5:**
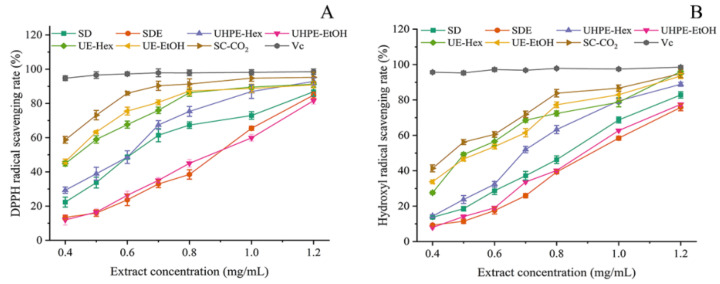
DPPH radical scavenging activity (**A**) and hydroxyl radical scavenging activity (**B**) of ILLTs extracted by different methods.

The ability of potential antioxidants to transfer an electron to a reducing compound can be assessed by calculating the DPPH free radical scavenging rate [14]. As shown in Figure 5A, the extracts obtained from the seven extraction methods (SD, SDE, UHPE-Hex, UHPE-EtOH, UE-EtOH, UE-Hex, and SC-CO_2_) all exhibited DPPH free radical scavenging ability, and this ability was concentration-dependent. Within the concentration range of 0.4–1.2 mg/mL, the ability of the UE-EtOH, UE-Hex, and SC-CO_2_ extracts to scavenge DPPH free radicals increased with the concentration of the *I*. *latifolius* leaf extract. At a concentration of 0.8 mg/mL, the DPPH free radical scavenging rates of the three extracts gradually approached that of the control Vc, with the DPPH free radical scavenging rate of SC-CO_2_ extract slightly higher than that of the UE-EtOH and UE-Hex extracts. However, the DPPH free radical scavenging ability of the SD, SDE, and UHPE-Hex extracts gradually increased with concentration, while the scavenging rate was consistently lower than that of the other three extracts. As illustrated in Figure 5B, the ability of the seven extracts to scavenge hydroxyl radicals gradually increased with concentration. Compared with SD, SDE, UHPE-Hex, UE-EtOH, and UE-Hex, the SC-CO_2_ extract exhibited a stronger ability to scavenge hydroxyl radicals.

**Figure 6:**
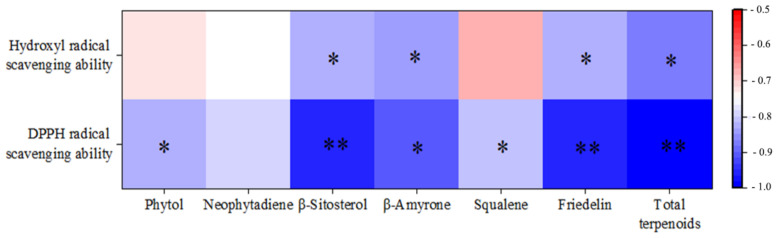
Correlation analysis of terpenoid content and antioxidant activity in *I. latifolius* leaf extracts. ** highly significant, *p* < 0.01; * significant, 0.01 < *p* < 0.05.

Studies have demonstrated that neophytadiene, phytol, β-sitosterol, β-amyrone, squalene, and friedelin, isolated from various plant organs, significantly contribute to the scavenging of DPPH free radicals, hydroxyl free radicals, superoxide anions, and carbon monoxide [22,36,37,38,39,40]. This highlights the remarkable antioxidant properties of these compounds across a range of reactive oxygen species. Thus, the correlation analysis conducted using SPSS revealed a significant association between the content of terpenoid compounds and antioxidant activity. As indicated in Figure 6, a significant correlation was observed between the two antioxidant methods (*p* < 0.05). Notably, there was an extremely significant negative correlation between the total content of terpenoids and the DPPH radical scavenging ability (IC_50_ value) (*p* < 0.01), as well as a significant negative correlation with the hydroxyl radical scavenging ability (IC_50_ value) (*p* < 0.05). Apart from neophytadiene, there was a highly significant (*p* < 0.01) or significant (*p* < 0.05) negative correlation between the DPPH radical scavenging ability (IC50 value) and the content of five terpenoid compounds. Meanwhile, a significant negative correlation was observed between the hydroxyl radical scavenging ability (IC50 value) and β-amyrone, β-sitosterol, and friedelin contents (*p* < 0.05). This suggested a potential synergistic effect among these compounds in enhancing antioxidant activity, warranting further investigation into their therapeutic potential.

### 3.5. Effects of Operating Conditions of SC-CO_2_ on the Extraction of ILLTs

**Figure 7:**
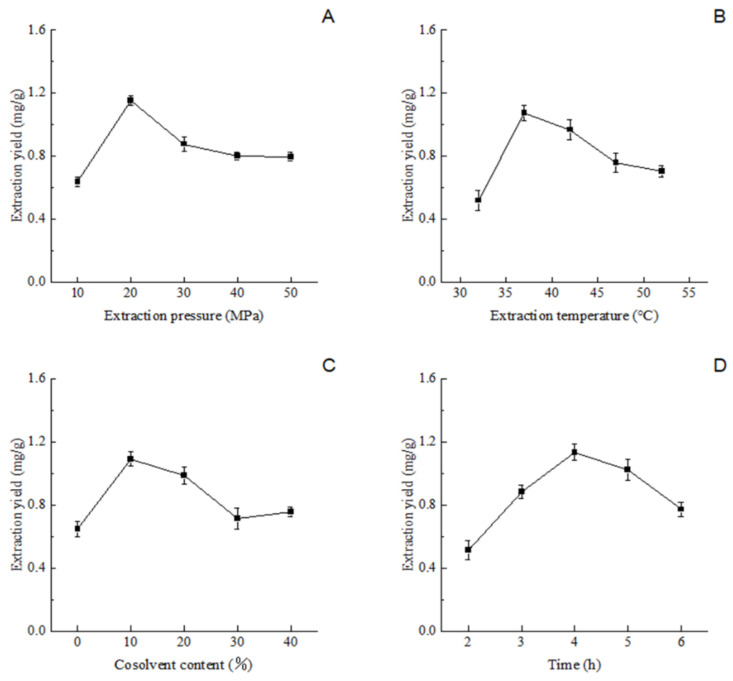
Effect of various factors on the extraction yield of ILLTs. Effect of extraction pressure (**A**), extraction temperature (**B**), cosolvent content (**C**), time (**D**) on extraction yield.

The results of all the single-factor experiments are presented in Figure 6. The extraction yield and selectivity of the compound are influenced by the density, diffusivity, and viscosity of the fluid [41]. As shown in Figure 7A, the yield of the ILLTs notably increased when the extraction pressure was increased from 10 MPa to 20 MPa. A higher pressure may facilitate the diffusion of the solute and its contact with the supercritical CO_2_, sequentially increasing the yield [42]. However, a pressure exceeding 20 MPa led to the reverse trend on account of the highly compressed CO_2_, resulting in solute–solvent repulsion, as reported by Gong et al. [16]. Therefore, the pressure range of 10–30 MPa was deemed more suitable for RSM studies.

As indicated in Figure 7B, the extraction rate of the ILLTs showed an initial increase followed by a decrease with the temperature rise, similar to the findings of [42]. Above 37 °C, the curve gently declined. Further increases in temperature might lead to the degradation of terpenoids [43], resulting in a decrease in extraction yield. Thus, the temperature range of 32–42 °C was chosen for the CCD design. 

Due to the nonpolar SC-CO_2_, polar modifiers facilitate the rapid and efficient solubility of polar molecules [44]. Initially, the yield of the ILLTs increased, reaching a maximum, but decreased when the cosolvent concentration exceeded 10% (*v*/*w*), as depicted in Figure 7C. According to González-Hernández et al. [45], less significant non-polar terpene hydrocarbons gradually transform into oxygenated terpenes with an increase in the cosolvent amount, demonstrating the cosolvent’s function. Subsequently, a cosolvent concentration of 10% (*v*/*w*) was selected as the CCD central point.

Under a constant extraction temperature (40 °C), extraction pressure (30 MPa), and ethanol content (10%, *v*/*w*), the impact of extraction time (2, 3, 4, 5, and 6 h) on ILLT yield was assessed. As shown in Figure 7D, the yield of the ILLTs increased with the extraction time until it reached 4 h, after which, a decreasing trend was observed. Gasparini et al. [46] discovered that longer extraction times and higher extraction pressures resulted in a decrease in diterpene selectivity, consistent with the findings mentioned above. Therefore, an extraction time of 4 h was selected as the central point of the CCD design.

### 3.6. Optimization of Extraction Conditions by CCD

The trial results and analysis of variance (ANOVA) of the experimental design tests are summarized in Table 4 and Table 5. The responses were fully explained by the predictive model established by the second-order polynomial Equation (8):

(8) $$Y=1.26+0.1431\;\times \;A+0.0621\;\times \;B+0.1070\;\times \;C+0.0549\;\times \;D+0.1035\;\times \;A\;\times \;B+\;0.0395\;\times \;A\;\times \;C+0.0457\;\times \;A\;\times \;D\;-\;0.0206\;\times \;B\;\times \;C+0.0245\;\times \;B\;\times \;D+0.0619\;\times \;C\;\times \;D\;-\;\;0{.2252\;\times \;A}^{2}-0{.1173\;\times \;B}^{2}+0{.008\;\times \;C}^{2}-\;0{.1708\;\times \;D}^{2}$$

The yields of ITLDs ranged from 0.6904 mg/g mg/g to 1.3107 mg/g. The determination coefficients of the models (R^2^) and the adjusted determination coefficient (R^2^_adj_) were 0.9738 and 0.9455, respectively, approaching 1, indicating that the model fit the experimental results [46]. The *p*-value of the model (<0.01) and the lack of fit *p*-value (>0.05) indicated that the model was statistically reliable [28]. The order of influence on the ILLT yield was as follows: extraction pressure > cosolvent concentration > extraction temperature > extraction time. Table 5 illustrates that the four linear coefficients (A, B, C, D), interaction coefficients (AB, AC, AD, CD), and quadratic term coefficients (A2, B2, D2) had significant effects on the yield of ITLDs (*p* < 0.05). 

**Figure 8:**
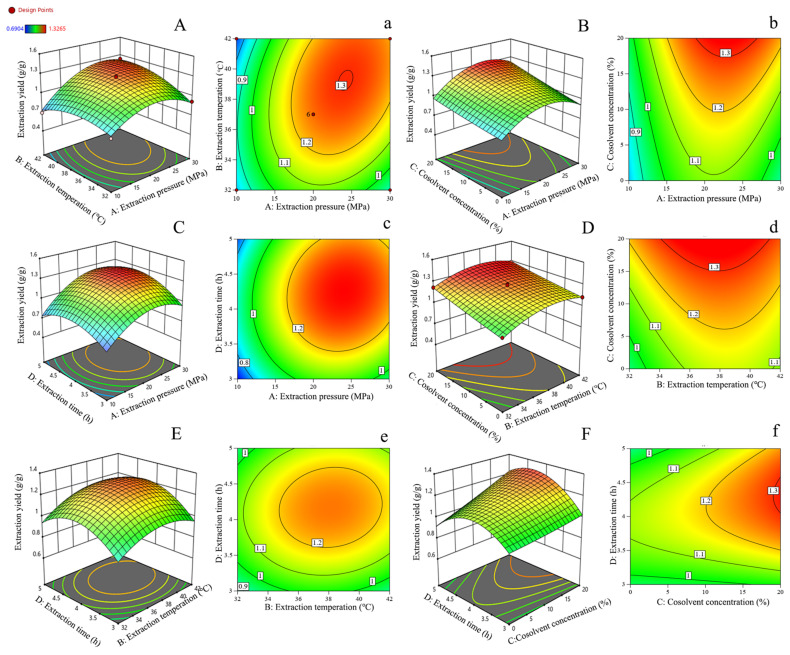
Response surface plots showing interactive effects of two-factor interactions on the yield of ITLDs. (**A**–**F**) contour map, (**a**–**f**) 3D surface map.

Figure 8 depicts the plots of the response surface and contour to more clearly reflect the influence relationship among the interactions of extraction temperature, extraction pressure, cosolvent concentration, and extraction time on the yield of ILLTs. As can be seen in Figure 8, the steepest slope of the AB response surface indicates that the interaction between extraction temperature and extraction pressure had the most significant impact on the extraction yield of ILLTs [47]. This result was consistent with the results of the ANOVA. The contour (Figure 8) indicates that the ILLT extraction yield initially improved and then declined with increasing extraction temperature, extraction pressure, or extraction time. Furthermore, there were positive effects of the cosolvent concentration on the extraction yield below 20%.

The response surface optimization model yielded the following optimal theoretical parameters: extraction pressure of 25.6 MPa, extraction temperature of 39.35 °C, cosolvent concentration of 20%, and extraction time of 4.52 h. Considering the experimental equipment conditions, the extraction time was set to 4.5 h, extraction pressure to 26 MPa, extraction temperature to 39 °C, and cosolvent concentration to 20%. Under these optimized conditions, the experimental yield of ILLTs was 1.44 ± 0.12 mg/g, which closely approximated the predicted value of 1.46 mg/g. Additionally, the yield of ITLDs extracted by supercritical CO_2_ fluid extraction was notably higher than that extracted by other techniques. In the research of Zulkafli et al. [19], β-siosterol, β-amyrene, and friedelin were likewise identified in bamboo leaf extracts using the SC-CO_2_ extraction method. Luca et al. [30] observed that lower pressures (90–110 bar) and lower temperatures (40–50 °C) contributed to improved extraction of the three hemp terpenes. A similar optimized condition (19 MPa/55 °C/5% EtOH) to that was employed in this study to enrich diterpenes from spent coffee grounds [17].

### 3.7. Purification Results of Terpenoid Compounds from I. latifolius Leaves

**Figure 9:**
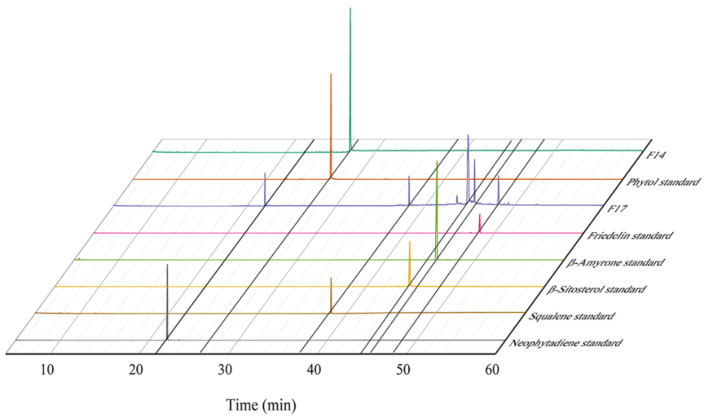
Gas chromatogram of purified samples (F14, F17) and standards.

The crude extract obtained using the optimized conditions underwent purification, and the purified samples were subsequently analyzed by GC-MS. Following purification through WelFlash C18-l, BUCHI-C18, and Sephadex LH-20, the crude extract of *I. latifolius* leaves exhibited an increased purity of components F14 and F17. The GC-MS results are presented in Figure 9. The gas chromatogram of the samples showed distinct peaks at 22, 27, 38, 44, 45, and 48 min, corresponding to standards for neophytadiene, phytol, β-sitosterol, β-amyrone, squalene, and friedelin. A comparison of the mass spectra of the six major compounds in the sample with those of known compounds and standards in the GC-MS NIST 20 library (Figures S1–S6) confirmed a high degree of agreement between the six major purified compounds and the standards. The purified F17 fraction also showed trace amounts of campesterol and stigmasterol, which are plant sterols belonging to triterpenoids, in addition to β-sitosterol. The purity of the crude extract of terpenoid compounds from *I. latifolius* leaves obtained under the optimized conditions increased from 12.91% to 93.34%.

### 3.8. Cytotoxicity Effect of ILLTs on HepG2 Cells

**Figure 10:**
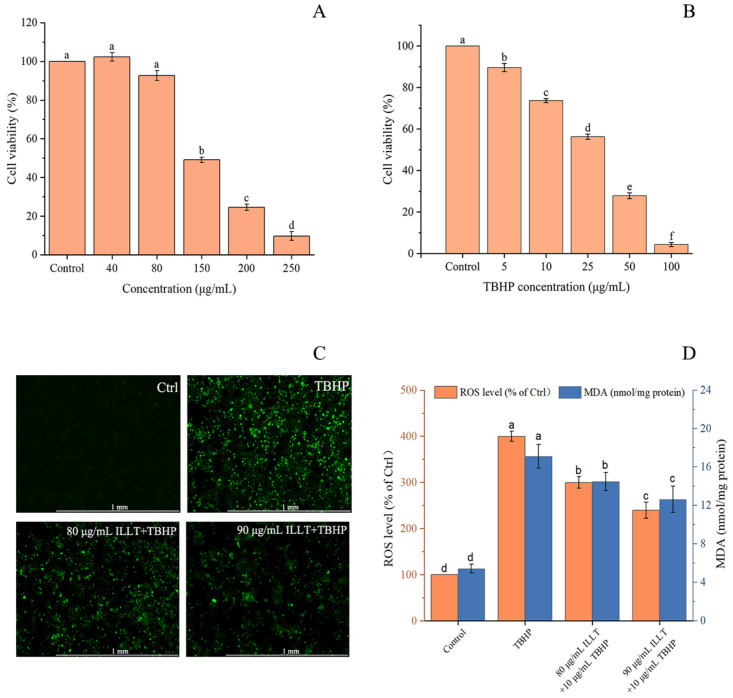
Effects of different concentrations of ILLTs (**A**) and TBHP (**B**) on cell viability of HepG2 cells; fluorescence images (**C**) of effects of different concentrations of ILLTs on TBHP-induced ROS formation in HepG2 cells; effects of different concentrations of ILLTs on TBHP-induced ROS and MDA production in HepG2 cells (**D**). Different letters indicate significant differences (*p* < 0.05).

High doses of plant extracts can be toxic and harmful to normal physiological systems. But, if the concentration is too low, it will not be effective [46]. Therefore, a toxicity analysis was performed to ensure that the extract was safe for the treatment of HepG2 cells. The direct effect of the ILLTs on HepG2 cell viability was studied in the concentration range of 0–250 μg/mL. The results in Figure 10A show that treatment with 80–250 μg/mL of the ILLTs for 24 h started to exhibit cytotoxicity in a dose-dependent manner (92.64–9.71%). However, at a concentration of 40 μg/mL, the cytotoxicity was negligible. Furthermore, the ILLTs exhibited an IC_50_ of 148.93 ± 9.93 μg/mL. Under the condition of a high cell number, we selected a concentration of 80–90 μg/mL for further experiments. Nguyen et al. [48] successfully isolated nine sesquiterpenes and two diterpenes from *Curcuma zedoaroides* (Zingiberaceae) rhizomes which displayed significant activity against A549, MCF-7, MDA-MB231, HL-60, and HepG2 cells, with IC_50_ values ranging from 3.13 μM to 30.10 μM. As depicted in Figure 10B, at a concentration of 10 g/mL of TBHP, the cell viability decreased to 73.67%, indicating that significant oxidative damage was induced by TBHP. Therefore, 10 mg/mL of TBHP was chosen as the experimental concentration to establish models of oxidative damage.

### 3.9. Effects of ILLTs on ROS and MDA Content of HepG2 Cells

Currently, there are little data on the antioxidant effects of plant terpenoids in HepG2 cells. To determine the protective effects of ILLTs against oxidative stress, the well-known inducer TBHP was used to generate excessive ROS and MDA levels in HepG2 cells. From the images shown in Figure 10D, TBHP increased the intracellular ROS level by 399.78% compared to the control group. Cells that were treated with 80 μg/mL and 90 μg/mL of the ILLTs significantly reduced the intracellular ROS concentration, which dropped by 299.70% and 239.61%, respectively. These findings were consistent with the fluorescence microscopy results (Figure 10C), where TBHP-stimulated cells exhibited intense green fluorescence due to ROS. 

ROS produced by cells can react with polyunsaturated fatty acids in biological membranes, leading to lipid peroxidation and the generation of MDA [49]. The groups treated with TBHP exhibited similar effects, regarding the accumulation of MDA (Figure 10D). When the cells were treated with 80–90 μg/mL of the ILLTs, the MDA content decreased by 15.31–26.07%. The data indicate that luteolin diminished the accumulation of intracellular ROS and MDA in a dose-dependent manner.

## 4. Conclusions

This study developed a rapid qualitative analysis method for the components of *I. latifolius* leaves and conducted a quantitative analysis of six major terpenoid compounds using GC-MS. A qualitative analysis was performed on crude extracts obtained from *I. latifolius* leaves using various extraction methods including SD, SDE, UHPE-Hex, UHPE-EtOH, UE-Hex, UE-EtOH, and SC-CO_2_ extraction methods. A total of 121 components were identified, with the total absolute contents of components from SD (18), SDE (28), UHPE-Hex (24), UHPE-EtOH (7), UE-Hex (42), UE-EtOH (27), and SC-CO_2_ (38) extracts being 10.360 mg, 7.347 mg, 30.686 mg, 4.682 mg, 35.957 mg,13.749 mg, and 28.160 mg, respectively. Neophytadiene, phytol, β-sitosterol, β-amyrone, squalene, and friedelin were identified as the main terpenoid compounds in *I. latifolius* leaves (ILLTs). The effects of the seven different extraction methods on the extraction rate, purity, and antioxidant activity of the ILLTs were investigated. The experimental results demonstrated that the SC-CO_2_ extraction method could significantly enhance the extraction rate and purity of terpenoid compounds from *I. latifolius* leaves. The SEM results also revealed substantial damage to the leaf structure caused by the SC-CO_2_ extraction method, facilitating solute solubilization. 

Additionally, the crude extract obtained by SC-CO_2_ exhibited significant advantages in terms of antioxidant activity. The correlation analysis suggested a potential synergistic effect among these compounds in enhancing antioxidant activity, warranting further investigation into their therapeutic potential. The optimal conditions for extracting ILLTs were determined through single-factor experiments and orthogonal experiments, resulting in an extraction time of 4.5 h, extraction pressure of 26 MPa, extraction temperature of 39 °C, and cosolvent concentration of 20%. Under these conditions, the actual extraction rate for the ILLTs reached 1.44 ± 0.12 mg/g, significantly higher than the other six methods. The ILLTs were demonstrated to have cytotoxic effects on HepG2 cells with an IC_50_ value of 148.93 ± 9.93 μg/mL. Moreover, the ILLTs were able to improve the cellular antioxidant status in a dose-dependent manner and protect HepG2 cells from oxidative damage by inhibiting ROS and MDA production. These terpenoid compounds have been shown to possess a potential synergistic effect in enhancing antioxidant activity, holding promising prospects for treating diseases caused by oxidative stress. In order to have a more comprehensive understanding of the processing properties and other functional properties of *Indocalamus latifolius* leaf terpenoids, further studies are needed.

## Figures and Tables

**Table 1:** DPPH free radical scavenging experiment reagent volumes.

Group	Samples, Vc	Ethyl Alcohol	DPPH Ethanol Solution (0.1 mmol/L)
A_sample_	1 mL	-	1 mL
A_blank_	1 mL	1 mL	-
A_control_	-	1 mL	1 mL

**Table 2:** The design and results of central composite design (CCD).

Factor	Units	Level of Factor		
		−1	0	1
A: Extraction stress	MPa	10	20	30
B: Extraction temperature	°C	32	37	42
C: Cosolvent	% (*v*/*w*)	0	10	20
D: Time	h	3	4	5

**Table 3:** Volatile composition of *I. latifolius* leaves expressed as the compound percentages based on GC-MS analysis.

No.	Compound	Molecular Formula	Molecular Weight	CAS	Absolute Content * (mg)								
					SD	SDE		UHPE-EtOH	UHPE-Hex	UE-EtOH		UE-Hex	SC-CO_2_
1	Isophytol	C_20_H_40_O	297	505-32-8	0.069 ± 0.001	0.036 ± 0.001							
2	Phytol	C_20_H_40_O	297	150-86-7	0.677 ± 0.043	0.438 ± 0.061		0.219 ± 0.017	1.553 ± 0.083	2.932 ± 0.096		5.38 ± 0.089	5.745 ± 0.073
3	Heneicosanol	C_21_H_44_O	313	3381-26-8								1.948 ± 0.101	0.086 ± 0.060
4	Octacosanol	C_28_H_58_O	411	557-61-9						0.148 ± 0.001		0.167 ± 0.001	0.071 ± 0.029
5	Lignocerol	C_24_H_50_O	355	506-51-4									0.094 ± 0.031
6	Campesterol	C_28_H_48_O	401	474-62-4						0.196 ± 0.037		0.346 ± 0.052	0.422 ± 0.047
7	Stigmasterol	C_29_H_48_O	413	83-48-7						0.104 ± 0.018		0.171 ± 0.046	0.269 ± 0.051
8	β-Sitosterol	C_29_H_50_O	415	5779-62-4					0.019 ± 0.006	3.094 ± 0.122		8.686 ± 0.141	9.909 ± 0.901
9	Lupeol	C_30_H_50_O	427	545-47-1									0.074 ± 0.061
10	2-(Octadecyloxy)-ethanol	C_20_H_42_O_2_	533	2136-72-3					0.013 ± 0.041				
11	2-Ethyl-1-decanol	C_12_H_26_O	186	21078-65-9					0.011 ± 0.001				
12	Tetrahydrofurfuryl alcohol	C_5_H_10_O_2_	102	97-99-4								0.061 ± 0.008	
13	1-Docosanol	C_22_H_46_O	326	661-19-8								0.285 ± 0.067	
14	Sclareol	C_20_H_36_O_2_	308	515-03-7						0.227 ± 0.001		0.084 ± 0.033	
15	(E, E)-10,12-Hexadecadien-1-ol acetat	C_16_H_30_O	238	765-19-5					0.033 ± 0.012				
16	Heptacosanol	C_27_H_56_O	397	2004-39-9						0.124 ± 0.092			
17	Nonacosanol	C_29_H_60_O	425	25154-56-7						0.279 ± 0.082			
18	Glutina-5-ene-3β-ol	C_30_H_50_O	427	545-24-4								0.072 ± 0.041	
19	Glycerin	C_3_H_8_O_3_	92	30918-77-5				12.36 ± 1.073					
20	Hexadecanal	C_16_H_32_O	240	629-80-1	0.064 ± 0.005	0.124 ± 0.076							
21	Octadecanal	C_18_H_36_O	268	638-66-4	4.050 ± 0.475	0.036 ± 0.018							0.041 ± 0.021
22	Hexanal	C_6_H_12_O	100	66-25-1		1.18 ± 0.089							
23	2-Hexenal	C_6_H_10_O	98	505-57-7								0.220 ± 0.074	
24	(E, E)-2,4-Heptadienal	C_7_H_10_O	110	4313-3-5		0.061 ± 0.023							
25	Benzeneacetaldehyde	C_8_H_8_O	120	122-78-1		1.648 ± 0.001							
26	Nonanal	C_9_H_18_O	142	124-19-6		0.916 ± 0.149							
27	β-Cyclocitral	C_10_H_16_O	152	432-25-7		0.102 ± 0.013							
28	(Z)-7-Tetradecenal	C_14_H_26_O	210	65128-96-3									0.127 ± 0.016
29	α-Ionone	C_13_H_20_O	192	127-41-3	0.003 ± 0.001	0.491 ± 0.165							0.024 ± 0.007
30	β-Apo-13-carotenone	C_18_H_26_O	258	17974-57-1						0.097 ± 0.011		0.072 ± 0.031	0.059 ± 0.023
31	2-Nonadecanone	C_19_H_38_O	282	629-66-3								0.057 ± 0.009	
32	Dotriacontanal	C_32_H_64_O	465	57878-00-9								2.016 ± 0.822	
33	2-Nonacosanone	C_29_H_58_O	423	17600-99-6								0.099 ± 0.021	
34	Phytone	C_18_H_36_O	268	502-69-2	0.709 ± 0.173					0.211 ± 0.054			0.032 ± 0.007
35	Trans-β-Ionone	C_13_H_20_O	192	14901-07-6					0.032 ± 0.008				0.070 ± 0.011
36	Hydroxyacetone	C_3_H_6_O_2_	74	116-09-6				9.461 ± 0.363					
37	D-Friedoolean-14-en-3-one	C_30_H_48_O	425	514-07-8						0.062 ± 0.013		0.296 ± 0.047	
38	4-(1,5-Dihydroxy-2,6,6-trimethylcyclohex-2-enyl) but-3-en-2-one	C_13_H_20_O_3_	208	38963-41-6									0.035 ± 0.014
39	4-(3-Hydroxybutyl)-3,5,5-trimethyl-2-cyclohexen-1-one	C_13_H_22_O_2_	210	36151-02-7									0.041 ± 0.016
40	β-Amyrone	C_30_H_48_O	425	638-97-1						0.517 ± 0.001		1.583 ± 0.891	1.591 ± 0.131
41	Lup-20(29)-en-3-one	C_30_H_48_O	425	1617-70-5								0.061 ± 0.012	0.071 ± 0.019
42	Friedelin	C_30_H_50_O	427	559-74-0					0.062 ± 0.018	1.405 ± 0.521		6.699 ± 1.091	4.100 ± 0.941
43	2,3-Dihydro-3,5-dihydroxy-6-methyl-4(H)-pyran-4-one	C_6_H_8_O_4_	144	28564-83-2				6.588 ± 0.873					
44	17-Pentatriacontene	C_15_H_30_O	226	2345-28-0					0.050 ± 0.016				
45	2-Methoxy-4-vinylphenol	C_9_H_10_O_2_	150	7786-61-0		0.256 ± 0.068							
46	3,5-Di-tert-butylphenol	C_14_H_22_O	206	1138-52-9								0.114 ± 0.070	
47	2,4-Di-t-butylphenol	C_14_H_22_O	206	96-76-4		0.059 ± 0.017							0.024 ± 0.013
48	2,2′-Methylenebis(6-tert-butyl-4-methylphenol)	C_23_H_32_O_2_	340	119-47-1							0.405 ± 0.026	0.452 ± 0.095	
49	2,6-Di-tert-butylphenol	C_14_H_22_O	206	128-39-2				0.561 ± 0.064					
50	γ-Tocopherol	C_28_H_48_O_2_	417	54-28-4							0.042 ± 0.015		0.080 ± 0.021
51	(Z, Z)-9,12-Octadecadienoic acid	C_20_H_36_O_2_	424	544-35-4									0.546 ± 0.088
52	5,6,7,7a-Tetrahydro-4,4,7a-trimethyl-2(4H)-benzofuranone	C_11_H_16_O_2_	180	15356-74-8									0.024 ± 0.006
53	Hexadecanoic acid, methyl ester	C_17_H_34_O_2_	270	112-39-0	0.132 ± 0.025							1.329 ± 0.471	0.357 ± 0.081
54	Ethyl palmitate	C_18_H_36_O_2_	284	628-97-7				0.096 ± 0.041			0.717 ± 0.092		
55	Octacosyl acetate	C_30_H_60_O_2_	452	18206-97-8	0.012 ± 0.001								
56	Methyl salicylate	C_8_H_8_O_3_	152	119-36-8			0.104 ± 0.019						
57	Benzyl salicylate	C_14_H_12_O_3_	228	118-58-1			0.036 ± 0.017						
58	1-Heneicosyl formate	C_22_H_44_O_2_	341	77899-03-7			0.013 ± 0.003						
59	(Z)-7-Hexadecenoic acid, methyl ester	C_17_H_32_O_2_	268	56875-67-3									0.074 ± 0.015
60	9-Hexadecenoic acid, ethyl ester	C_18_H_34_O_2_	282	54546-22-4								0.118 ± 0.027	
61	Heptadecanoic acid, ethyl ester	C_19_H_38_O_2_	299	14010-23-2							0.044 ± 0.009	0.046 ± 0.011	
62	Elaidic acid ethyl ester	C_20_H_38_O_2_	311	6114-18-7								0.763 ± 0.039	
63	Ethyl icosanoate	C_22_H_44_O_2_	341	18281-05-5							0.060 ± 0.017	0.103 ± 0.005	
64	1-Hexadecanol, acetate	C_18_H_36_O_2_	284	629-70-9								0.072 ± 0.033	
65	Butyrolactone	C_4_H_6_O_2_	86	3068-88-0				1.402 ± 0.107					
66	Ethyl stearate	C_20_H_40_O_2_	313	111-61-5									0.035 ± 0.007
67	Methyl octadeca-9,12-dienoate	C_19_H_34_O_2_	294	2566-97-4									0.162 ± 0.017
68	1,2,3-Trielaidoyl glycerol	C_57_H_104_O_6_	885	537-39-3									0.496 ± 0.065
69	Phytyl acetate	C_22_H_42_O_2_	339	10236-16-5									0.044 ± 0.013
70	β-Sitosterol acetate	C_31_H_52_O_2_	457	915-05-9									0.086 ± 0.014
71	α-Tocopheryl acetate	C_31_H_52_O_3_	473	7695-91-2									0.059 ± 0.005
72	Ethyl linolenate	C_20_H_34_O_2_	306	1191-41-9							1.007 ± 0.085		
73	Linoleic acid ethyl ester	C_20_H_36_O_2_	309	544-35-4							0.579 ± 0.053	0.702 ± 0.074	
74	2,2-Dimethyl-3-(3,7,12,16,20-pentamethyl-3,7,11,15,19-heneicosapentaenyl)-oxirane	C_30_H_50_O	427	7200-26-2								0.053 ± 0.006	0.056 ± 0.011
75	1-Pentadecene	C_15_H_30_	210	13360-61-7									0.130 ± 0.025
76	(E)-3-Eicosene	C_20_H_40_	281	74685-33-9					0.077 ± 0.012				
77	(E)-5-Eicosene	C_20_H_40_	281	74685-30-6					0.159 ± 0.034				
78	1-Docosene	C_22_H_44_	309	1599-67-3			0.084 ± 0.013		0.433 ± 0.058				
79	Squalene	C_30_H_50_	411	111-02-4			0.006 ± 0.001				0.075 ± 0.009	0.054 ± 0.005	0.144 ± 0.012
80	1,19-Eicosadiene	C_20_H_38_	279	14811-95-1			0.020 ± 0.001		0.380 ± 0.016				
81	8-Heptadecene	C_17_H_34_	238	2579-4-6			0.438 ± 0.081						
82	7-Methyl-6-Tridecene	C_14_H_28_	196	24949-42-6			0.149 ± 0.036						
83	(Z)-9-Tricosene	C_23_H_46_	323	27519-02-4			0.246 ± 0.056						
84	Neophytadiene	C_20_H_38_	279	504-96-1	0.002 ± 0.001		0.016 ± 0.002		0.383 ± 0.027		0.633 ± 0.036	0.073 ± 0.008	0.122 ± 0.015
85	Nonadecane	C_19_H_40_	269	629-92-5	0.247 ± 0.062				0.027 ± 0.004				
86	Heptacosane	C_27_H_56_	381	593-49-7	0.080 ± 0.017				0.197 ± 0.016				
87	Triacontane	C_30_H_62_	423	638-68-6	0.224 ± 0.078								
88	Heneicosane	C_21_H_44_	297	629-94-7	1.960 ± 0.106		0.099 ± 0.012		0.187 ± 0.009			0.289 ± 0.017	
89	Pentacosane	C_25_H_52_	353	629-99-2			0.036 ± 0.011		0.050 ± 0.007		0.342 ± 0.029	0.209 ± 0.071	
90	Tricosane	C_23_H_48_	325	638-67-5	0.062 ± 0.008		0.443 ± 0.058						
91	Undecane	C_11_H_24_	156	1120-21-4									
92	Hexadecane	C_16_H_34_	226	544-76-3								0.034 ± 0.016	
93	Docosane	C_22_H_46_	311	629-97-0								0.027 ± 0.002	
94	Tetratetracontane	C44H_90_	619	7098-22-8								0.049 ± 0.007	
95	Hentriacontane	C_31_H_64_	437	630-04-6								1.074 ± 0.006	
96	2-Methylhexacosane	C_27_H_56_	381	1561-02-0								0.292 ± 0.021	
97	3,5,24-Trimethyl-tetracontane	C_43_H_88_	605	55162-61-3					0.017 ± 0.008				
98	2,6,10,15-Tetramethyl-heptadecane	C_21_H_44_	297	54833-48-6					0.013 ± 0.002				
99	5,14-Dibutyloctadecane	C_26_H_54_	367	55282-13-8					0.022 ± 0.002				
100	7-Hexyltridecane	C_19_H_40_	269	7225-66-3	1.458 ± 0.075				0.022 ± 0.003				
101	8-Hexyl-pentadecane	C_21_H_44_	297	13475-75-7	0.499 ± 0.035		0.210 ± 0.048						
102	Dotriacontane	C_32_H_66_	451	544-85-4	0.049 ± 0.006								
103	Tetracosane	C_24_H_50_	339	646-31-1	0.062 ± 0.014		0.084 ± 0.022		0.044 ± 0.006				0.215 ± 0.061
104	Octadecane	C_18_H_38_	254	593-45-3			0.016 ± 0.003		0.192 ± 0.047				
105	2,6,10,15-Tetramethyl heptadecane	C_21_H_44_	297	54833-48-6							0.054 ± 0.007		
106	Phytonadione	C_31_H_46_O_2_	451	84-80-0							0.092 ± 0.011		
107	Cholesterol	C_27_H_46_O	387	57-88-5							0.136 ± 0.038		
108	2-Palmitoylglycerol	C_19_H_38_O_4_	331	23470-00-0								0.304 ± 0.068	
109	Hexadecanamide	C_16_H_33_NO	255	629-54-9								0.968 ± 0.056	
110	(Z)-9-Octadecenamide	C_18_H_35_NO	281	301-02-0					0.706 ± 0.090			0.084 ± 0.029	0.115 ± 0.024
111	Vitamin E	C_29_H_50_O_2_	431	2074-53-5							0.166 ± 0.012	0.448 ± 0.034	0.531 ± 0.078

* Absolute content means the content in 20 g of raw material.

**Table 4:** Response surface experiment results.

Run	A ^1^	B ^1^	C ^1^	D ^1^	Yield of Diterpenes (mg/g)	
					Experimental	Predicted
1	0	0	0	0	0.75	0.76
2	−1	−1	0	0	0.28	0.31
3	0	0	1	−1	0.58	0.57
4	0	0	−1	−1	0.49	0.48
5	0	0	0	0	0.77	0.76
6	1	−1	0	0	0.42	0.40
7	1	1	0	0	0.74	0.72
8	−1	1	0	0	0.19	0.23
9	0	0	−1	1	0.47	0.47
10	0	0	1	1	0.81	0.80
11	1	0	0	1	0.60	0.61
12	0	0	0	0	0.78	0.76
13	−1	0	0	−1	0.22	0.21
14	0	1	−1	0	0.63	0.61
15	0	1	1	0	0.78	0.78
16	0	−1	−1	0	0.47	0.44
17	1	0	0	−1	0.40	0.40
18	−1	0	0	1	0.24	0.23
19	0	0	0	0	0.72	0.76
20	0	−1	1	0	0.72	0.70
21	1	0	−1	0	0.52	0.52
22	−1	0	1	0	0.49	0.45
23	0	0	0	0	0.75	0.76
24	0	0	0	0	0.77	0.76
25	−1	0	−1	0	0.35	0.31
26	0	1	0	−1	0.47	0.45
27	0	−1	0	1	0.43	0.44
28	0	−1	0	−1	0.37	0.38
29	1	0	1	0	0.83	0.81
30	0	1	0	1	0.63	0.61

^1^ A, extraction stress; B, extraction temperature; C, cosolvent concentration; D, extraction time.

**Table 5:** ANOVA for response surface quadratic model.

Source	Sum of Squares	df	Mean Square	F-Value	*p*-Value
Model	1.09	14	0.0777	117.88	<0.0001 **
A	0.2458	1	0.2458	372.87	<0.0001 **
B	0.0464	1	0.0464	70.32	<0.0001 **
C	0.1374	1	0.1374	208.49	<0.0001 **
D	0.0361	1	0.0361	54.84	<0.0001 **
AB	0.0428	1	0.0428	64.97	<0.0001 **
AC	0.0062	1	0.0062	9.48	0.0081
AD	0.0084	1	0.0084	12.7	0.0031 *
BC	0.0017	1	0.0017	2.58	0.1271
BD	0.0024	1	0.0024	3.66	0.0744
CD	0.0153	1	0.0153	23.21	0.0003
A^2^	0.3478	1	0.3478	527.59	<0.0001 **
B^2^	0.0943	1	0.0943	143.02	<0.0001 **
C^2^	0.0004	1	0.0004	0.6609	0.4245
D^2^	0.1999	1	0.1999	303.31	<0.0001 **
Residual	0.0086	13	0.0007		
Lack of Fit	0.0079	10	0.0008	3.8	0.2791
Pure Error	0.0006	3	0.0002		
Cor Total	1.1	29		117.88	
R^2^ = 0.9738 R^2^_adj_ = 0.9455					2.560 × 10^−4^

** highly significant, *p* < 0.01; * significant, 0.01 < *p* < 0.05.

## Data Availability

The original contributions presented in the study are included in the article/Supplementary Materials, further inquiries can be directed to the corresponding authors.

## Supplementary Materials

The following supporting information can be downloaded at: https://www.mdpi.com/article/10.3390/foods13111719/s1, Figure S1: Mass spectrum of purified samples and standards of neophytadiene; Figure S2: Mass spectrum of purified samples and standards of phytol; Figure S3: Mass spectrum of purified samples and standards of squalene; Figure S4: Mass spectrum of purified samples and standards of β-amyrone; Figure S5: Mass spectrum of purified samples and standards of β-sitosterol; Figure S6: Mass spectrum of purified samples and standards of friedelin.
