# Supplementary material for: Supercritical CO2 Extraction of Terpenoids from Indocalamus latifolius Leaves: Optimization, Purification, and Antioxidant Activity
Source: Foods. 2024 May 30;13(11):1719. doi: 10.3390/foods13111719 (PMC11171701; doi:10.3390/foods13111719)
Supplement: Supplementary file 1 [file foods-13-01719-s001.zip › foods-3015492-supplementary.pdf]

Supplementary Materials: Figure S1: Mass spectrum of neophytadiene purified samples and standards; Figure S2: Mass spectrum of phytol purified samples and standards; Figure S3: Mass spectrum of squalene purified samples and standards; Figure S4: Mass spectrum of β-amyrone purified samples and standards; Figure S5: Mass spectrum of β-sitosterol purified samples and standards; Figure S6: Mass spectrum of friedelin purified samples and standards
