# Supplementary figures and images for: Supercritical CO2 Extraction of Terpenoids from Indocalamus latifolius Leaves: Optimization, Purification, and Antioxidant Activity
Source: Foods. 2024 May 30;13(11):1719. doi: 10.3390/foods13111719 (PMC11171701; doi:10.3390/foods13111719)

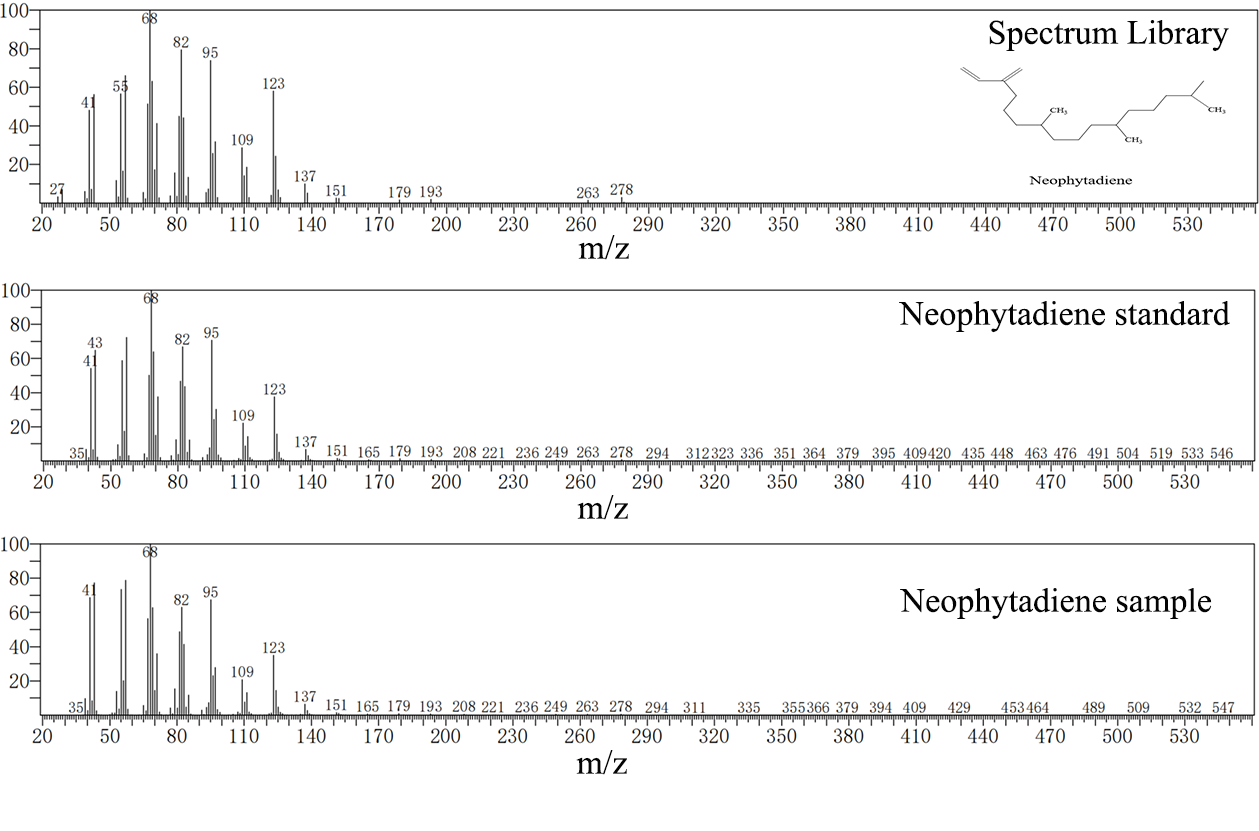

Supplement: Supplementary file 1 [file foods-13-01719-s001.zip › Figure S1.tif]

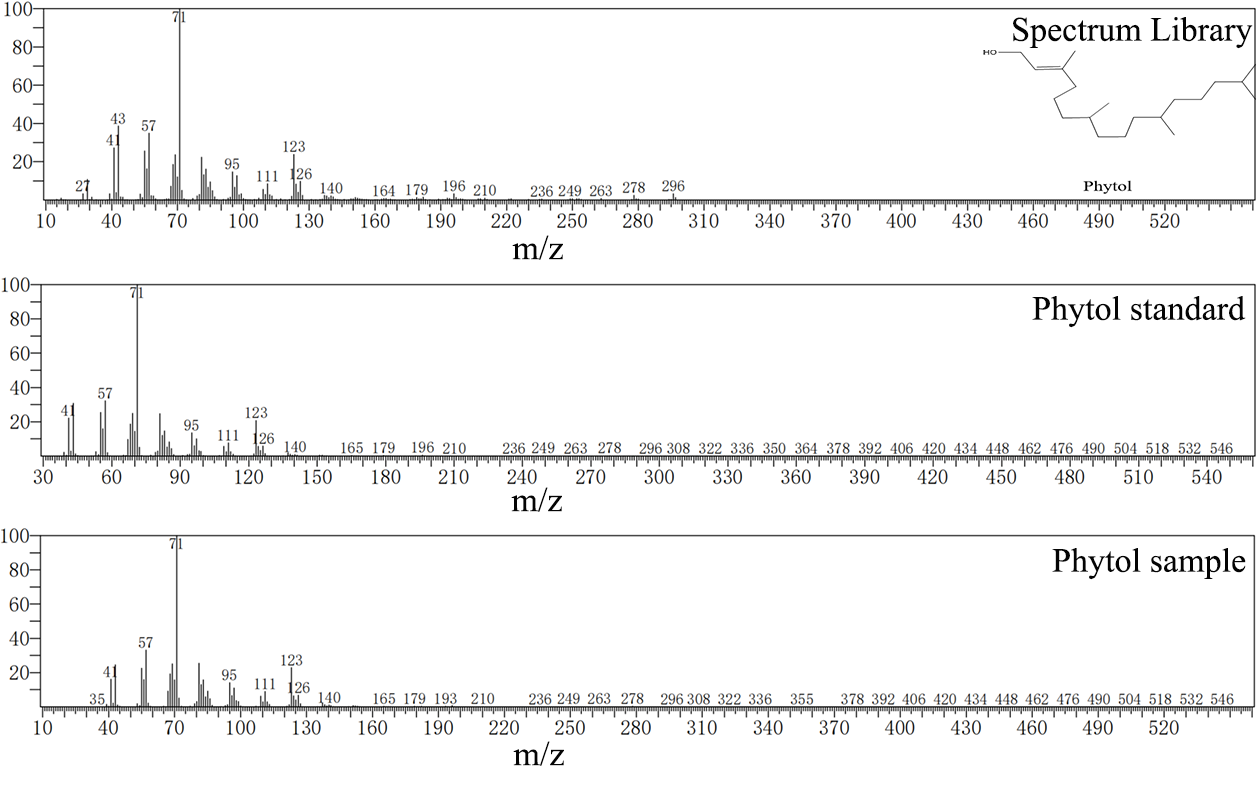

Supplement: Supplementary file 1 [file foods-13-01719-s001.zip › Figure S2.tif]

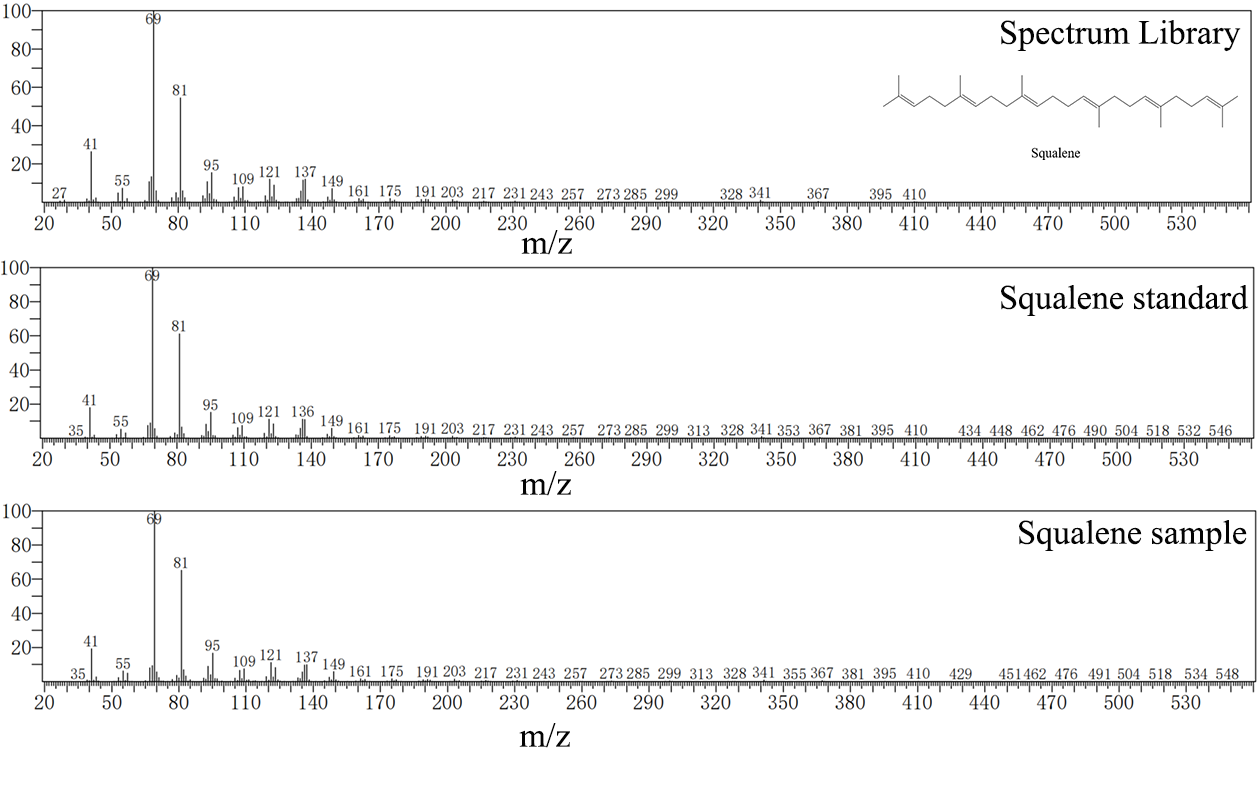

Supplement: Supplementary file 1 [file foods-13-01719-s001.zip › Figure S3.tif]

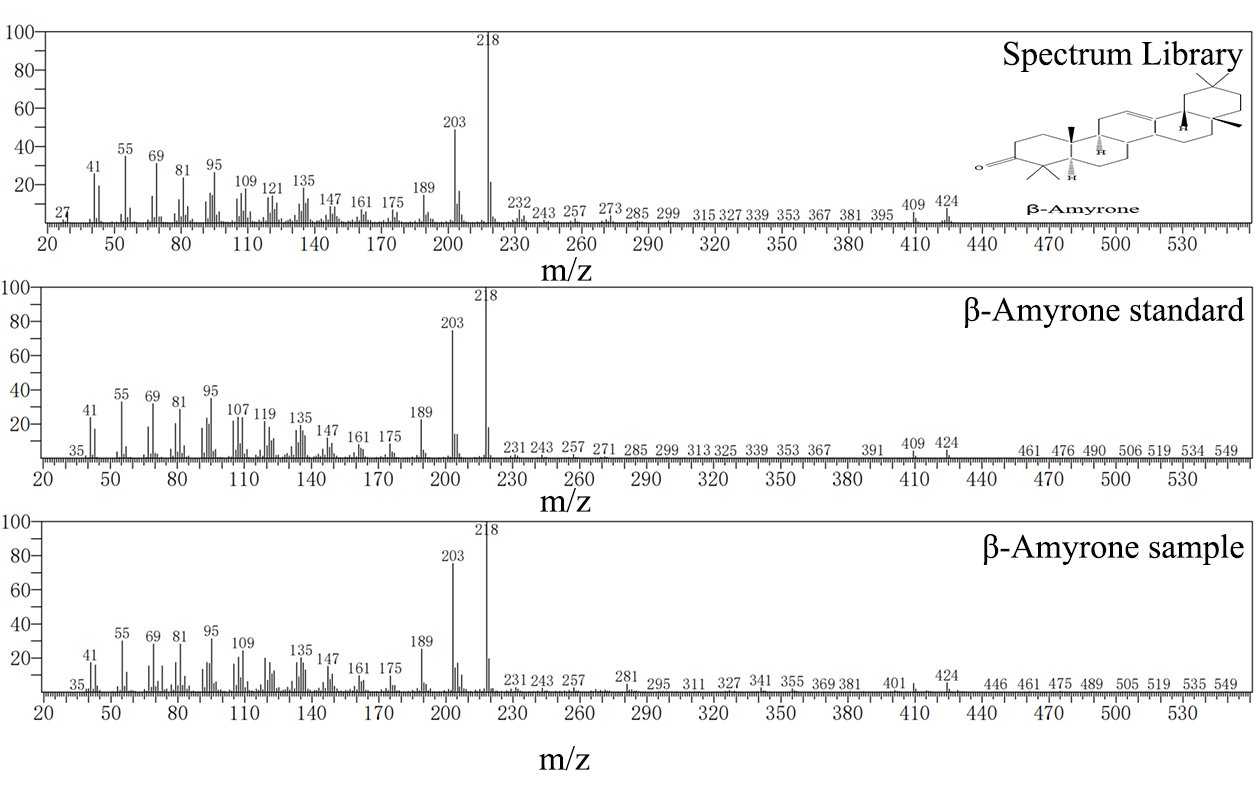

Supplement: Supplementary file 1 [file foods-13-01719-s001.zip › Figure S4.tif]

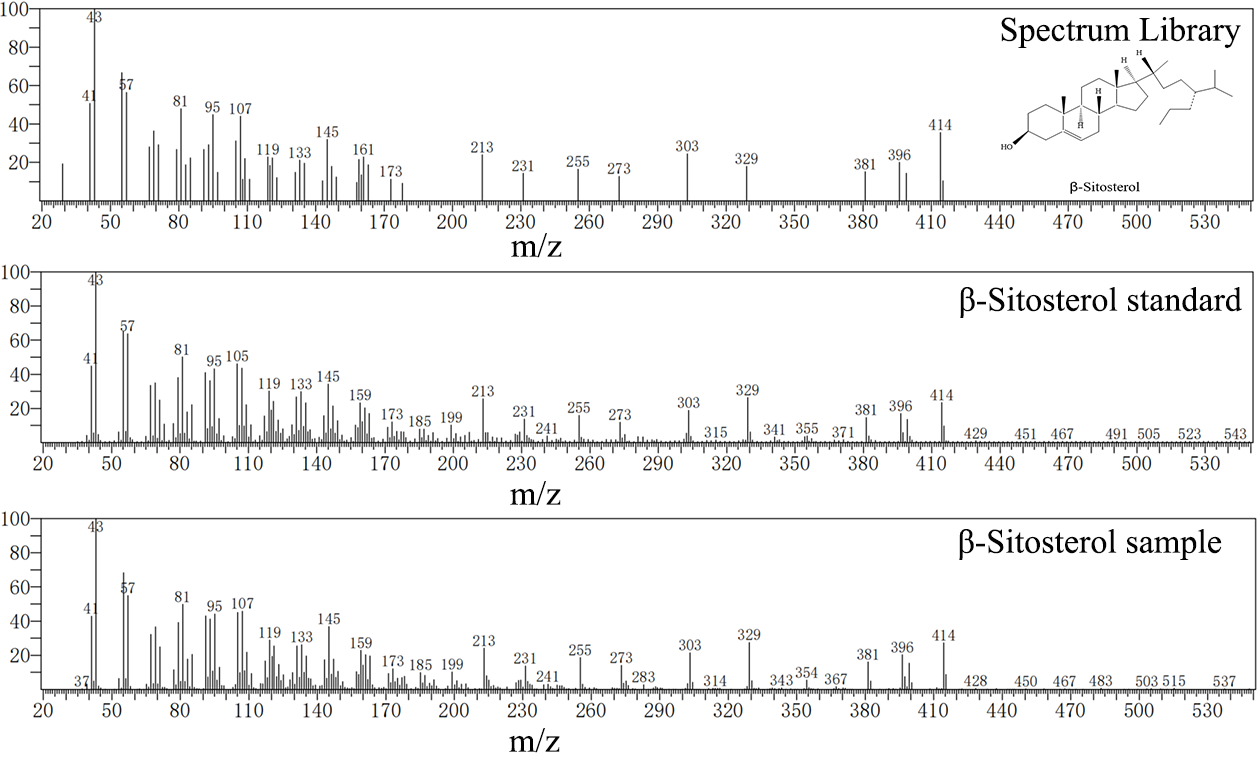

Supplement: Supplementary file 1 [file foods-13-01719-s001.zip › Figure S5.tif]

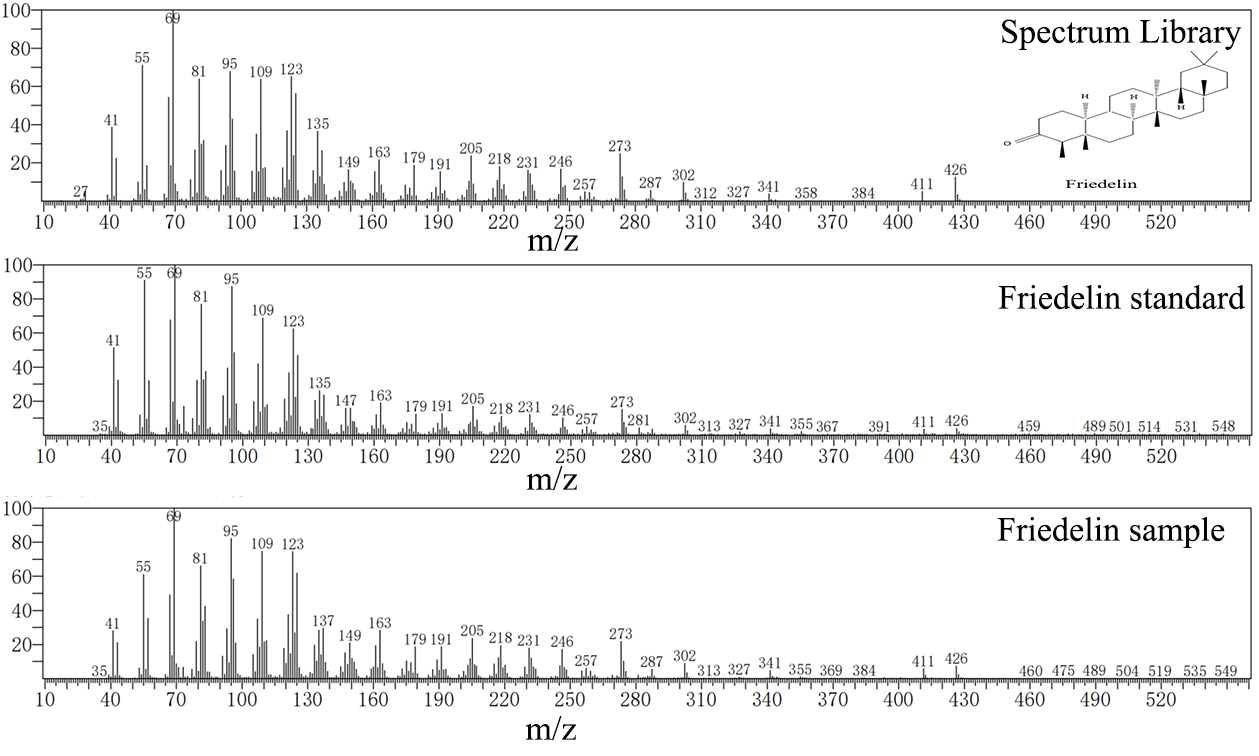

Supplement: Supplementary file 1 [file foods-13-01719-s001.zip › Figure S6.tif]
